# Controlling the Dissolution Behavior of (Meth)acrylate-Based Photoresist Polymers in Tetramethylammonium Hydroxide by Introducing Adamantyl Groups

**DOI:** 10.3390/ma18020381

**Published:** 2025-01-15

**Authors:** Jinyoung Kim, Choong-Jae Lee, Dong-Gun Lee, Geon-Ho Lee, Jayoung Hyeon, Yura Choi, Namchul Cho

**Affiliations:** Department of Energy Engineering, Soonchunhyang University, 22 Soonchunhyang-ro, Asan 31538, Republic of Korea; kjy1624@sch.ac.kr (J.K.); cndwolee1397@sch.ac.kr (C.-J.L.); alclsro6202@naver.com (D.-G.L.); sinb0603@sch.ac.kr (G.-H.L.); jayoung@sch.ac.kr (J.H.); bnbn3238@sch.ac.kr (Y.C.)

**Keywords:** (meth)acrylate-based polymer, aliphatic monomer, aqueous alkaline developer, solubility

## Abstract

(Meth)acrylate polymers are commonly used as photoresist materials in photolithography. However, these polymers encounter the problem of swelling during the development process. To address this, we explored the use of a hydrophobic group to control the solubility in the hydrophilic developer. In this study, we synthesized two types of polymers to evaluate the impact of the developer on (meth)acrylate polymers for photoresist applications. Adamantyl methacrylate (AdMA) was selected as the hydrophobic group, while 2-ethoxyethyl acrylate (2-EEA) served as the hydrophilic group, enabling the synthesis of both hydrophilic and hydrophobic polymers. Our goal was to assess how the presence of adamantyl monomers influenced the solubility of the polymer. This study demonstrated that solubility was primarily influenced by functional groups, particularly hydrophobic groups, rather than other factors. Polymers with more than 50% hydrophobic groups can be effectively controlled for their solubility in TMAH. These findings show that the solubility of photoresist polymers in TMAH can be tuned by incorporating a high proportion of hydrophobic groups. The study further confirms the role of adamantyl monomers as effective hydrophobic (aliphatic) groups in modulating the solubility of (meth)acrylate polymers in developer solutions.

## 1. Introduction

Chemically amplified resists, introduced by Ito and Wilson in 1982, have been improved to offer excellent patterning performance and high sensitivity [1,2,3]. Recently, ArF photoresists have been used to develop patterns with features smaller than 50 nm, and a variety of polymers for photoresists (PRs) have been designed to enhance pattern quality [4,5,6,7,8,9,10,11]. Specifically, at the 193 nm wavelength of the ArF excimer laser, (meth)acrylate polymers exhibit good transmittance. In contrast, polymers designed for other wavelengths, such as those containing aromatic or melamine structures, show lower transmittance. As a result, the deprotection process of (meth)acrylate polymers has been extensively studied using a 193 nm light source [1,12,13,14,15]. These polymers consist of multiple monomers with various functionalities. The polar group is soluble in an aqueous alkaline developer and ensures adhesion to the substrate, while acid-labile groups remain insoluble in the developer until they undergo deprotection upon exposure. Additionally, aliphatic ring groups enhance the etching resistance and thermal stability of (meth)acrylate polymers [16,17,18,19,20,21].

In the (meth)acrylate polymers used for photoresists, the polar group presents a potential issue. Specifically, the aqueous alkaline developer used in the development process can cause polymer swelling owing to interactions with the hydroxyl groups [22,23]. To address this, swelling has been mitigated by cross-linking the polymers [24,25]. Epoxy functional groups [26] and melamine crosslinkers [27] have been investigated for cross-linking (meth)acrylate polymers, but they are not suitable for use with a 193 nm light source. To overcome this limitation, transesterification has been explored as a novel approach [24,25]. Transesterification is a cross-linking reaction with hydroxyl groups [28]. In this case, the polymer has a structure suitable for a 193 nm light source. These cross-linked polymers can form negative-tone patterns because the exposed portions do not dissolve in the developer. In contrast, when the solubility of polymers in TMAH cannot be controlled by functional groups, the polymers will dissolve in the developer, even if they are cross-linked. In this case, the polymer dissolves in TMAH upon exposure [22]. Therefore, the aliphatic groups in the polymer play a key role in controlling its solubility in the developer, with the hydrophobicity of these groups counteracting the hydrophilicity of TMAH [22,25]. However, the aliphatic groups previously used in these polymers were limited to the sole function of controlling polymer solubility [25]. Polymers used for photoresists must possess multiple functions to improve pattern quality. To address these limitations, we incorporated an adamantyl monomer, which has been explored in other (meth)acrylate polymers for photoresists. While adamantyl monomers are also aliphatic groups, studies have shown that they allow polymers to exhibit a range of functions when combined with additional functional groups [17].

In this study, we synthesized a polymer incorporating an adamantyl monomer as an aliphatic (hydrophobic) group to explore its potential for modulating the polymer’s solubility. Additionally, we synthesized another polymer using an ethoxy group, which exhibited properties opposite to those of a hydrophilic group, to compare the solubility control mechanism of hydrophobic groups. For this, we used hydroxypropyl methacrylate (HPMA) and tert-butyl methacrylate (t-BMA) as the polar group and acid-labile group, respectively. Moreover, 1-adamantyl methacrylate (AdMA) was used as the hydrophobic group, and 2-ethoxyethyl acrylate (2-EEA) was chosen as the hydrophilic group to synthesize two types of polymers with considerably different solubilities in the aqueous alkaline developer (tetramethylammonium hydroxide, TMAH). When 2-EEA was used, an increase in solubility was observed. In contrast, the incorporation of AdMA resulted in lower solubility. This confirms that a polymer with a high ratio of AdMA can effectively control solubility in TMAH. As a result, this polymer is well-suited for negative photoresists involving transesterification. The study further confirms that adamantyl monomers can serve as effective aliphatic groups for regulating the solubility of (meth)acrylate polymers in developer solutions. Therefore, constructing a polymer with a high proportion of AdMA enables control over its solubility in TMAH, making it suitable for negative photoresist applications with transesterification. Overall, these findings demonstrate that adamantyl monomers can play a key role in modulating the solubility of (meth)acrylate polymers in developing solutions.

## 2. Materials and Methods 

### 2.1. Materials 

Hydroxypropyl methacrylate (HPMA), propylene glycol monomethyl ether acetate (PGMEA), benzoyl peroxide (BPO), and tetramethylammonium hydroxide (TMAH) were purchased from Sigma-Aldrich (St. Louis, MO, USA). N-((trifluoromethylsulfonyl)oxy)-5-norbornene-2,3-dicarboximide (NDI) was obtained from Alfa Aesar (Ward Hill, MA, USA). Heptane (95%), ethyl acetate (EA, 99.5%), and isopropanol (IPA, 99.5%) were purchased from Samchun Chemical (Seoul, Republic of Korea). Acetone (99.5%) and n-hexane (95%) were purchased from Deajung Chemical (Siheung, Republic of Korea). 1-Adamantyl methacrylate (AdMA) and tert-butyl methacrylate (t-BMA) were acquired from TCI (Tokyo, Japan). 2-Ethoxyethyl acrylate (2-EEA) was purchased from AmBeed (Arlington Hts, IL, USA).

### 2.2. Synthesis of HPMA, t-BMA, AdMA (HTA) Copolymer 

HTA copolymers were synthesized following the procedure outlined below. HPMA (4.33 g), AdMA (6.61 g), and t-BMA (5.69 g) were combined as the monomers in a beaker. BPO (0.42 g) was used as the initiator, and PGMEA (83.15 g) served as the solvent. The mixture was stirred for 30 min at room temperature. Next, PGMEA (41.58 g) was added to a batch reactor, which was then heated to 85 °C. The monomer and initiator mixture was injected into the reactor and allowed to react for 3 h, followed by an additional 16 h of reaction time. ^1^H NMR (CDCl_3_, 400 MHz): δH 4.80 (1H, br), 4.05 (1H, br), 3.60 (2H, br), 3.36 (2H, s), 2.10 (2H, m), 1.80 (2H, br), 1.60 (2H, s), 1.43 (9H, s), 1.23 (2H, m), 1.02 (3H, br). M_n_ = 10,090–11,925, *Đ* = 1.82–1.59.

After the reaction, the precipitation step was performed. The reaction mixture was slowly injected dropwise into heptane (8 times the volume of the mixture) and stirred for 30 min. The mixture was then allowed to stand for 12 h for precipitation. Afterward, heptane was evaporated, and the precipitate was dried under a vacuum to obtain the polymer.

The polymer was purified by extraction to remove low molecular weight oligomers. HTA (5 g) was dissolved in EA (200 mL), and the mixture was washed with DIW (200 mL). This washing procedure was repeated 10 times. After the final wash, EA was evaporated, and the purified polymer was obtained by drying the polymer slurry under a vacuum.

### 2.3. Synthesis of HTE Copolymer 

HTE copolymers were synthesized following the procedure outlined below. HPMA (4.33 g), 2-EEA (5.11 g), and t-BMA (5.69 g) were added to a beaker as the monomers. BPO (0.38 g) was used as the initiator, and PGMEA (75.65 g) served as the solvent. The mixture was stirred for 30 min at room temperature. Subsequently, PGMEA (37.83 g) was transferred to a batch reactor and heated to 85 °C. The mixture of monomers and initiator was injected into the batch reactor, where the reaction proceeded for 3 h. The reaction was then continued for an additional 16 h. ^1^H NMR (CDCl_3_, 400 MHz): δH 4.80 (1H, br), 4.05 (1H, br), 3.75 (2H, br), 3.39 (2H, s), 3.30 (3H, s), 2.45 (2H, s), 1.74 (2H, br), 1.60 (2H, s), 1.19 (2H, m), 1.05 (3H, br), 0.85 (2H, br). M_n_ = 11,028–12,605, *Đ* = 1.74–1.67.

The reaction mixture was added dropwise to heptane (8:1 volume ratio) and stirred for 30 min. After allowing the mixture to stand for 12 h to ensure precipitation, heptane was evaporated, and the polymer was vacuum-dried to yield the final product. The polymer was then purified by extraction to remove low molecular weight oligomers. HTE (5 g) was dissolved in EA (200 mL), and the mixture was washed with DIW (200 mL). This washing process was repeated 10 times. Afterward, EA was evaporated, and the purified polymer was obtained by drying the polymer slurry under a vacuum.

### 2.4. Preparation of Patterns

The photoresist was prepared following the method of Asakura et al. [29] (PGMEA:NDI:Polymer (HTA, HTE) = 600:2:100, wt.%). To prepare the surface, the Si wafer was cleaned with acetone and IPA, then treated with O_3_ plasma using a vacuum plasma system (CUTE, Pemto Science, Hwaseong-si, Republic of Korea). The Si wafer was then spin-coated with the photoresist at 3000 rpm for 240 s. After coating, the wafer was annealed at 100 °C for 60 s. For the exposure step, the mask was aligned to the annealed wafer and exposed to a 365 nm light source for 300 s. In the postexposure bake step, the hot plate temperature was set to 120 °C, and the patterned wafer was baked for 60 s. Finally, the annealed wafer was dipped in a developer solution (TMAH, 0.26 × 10^−2^ N) for 15 s and then baked again at 100 °C for 60 s.

### 2.5. Characterization 

Proton nuclear magnetic resonance (^1^H NMR) measurements were performed using a 400 MHz Ascend 400 spectrometer (Bruker, Middlesex County, MA, USA) with CDCl_3_ as the solvent and 64 scans. Molecular weight distributions were determined by gel permeation chromatography (GPC, Waters, Worcester County, MA, USA) using THF as the mobile phase. The mobile phase flow rate was set to 0.1 mL min^−1^, and the GPC system was equipped with KS-801, KS-802, and KS-803 columns, along with a differential refractive index (RI 804) detector. X-ray diffraction (XRD) spectra were collected using a Rigaku MiniFlex diffractometer (Tokyo, Japan) in the 2*θ* range of 2°–50°. FT–IR spectra were recorded using a FT-200 spectrophotometer (Horiba, Kyoto, Japan). Static water contact angle measurements were performed with a drop shape analyzer (DSA30S, KRÜSS GmbH, Hamburg, Germany) under ambient conditions of 25 °C and 16% relative humidity. Pattern images were captured using an optical microscope (BX53M, OLYMPUS, Shinjuku, Japan) at various magnifications. The film weight, including the solvent, was measured with an analytical balance (PIONEERTM PX124M, OHAUS, Parsippany, NJ, USA).

## 3. Results and Discussion 

### 3.1. Preparation of Copolymers 

**Scheme 1:**
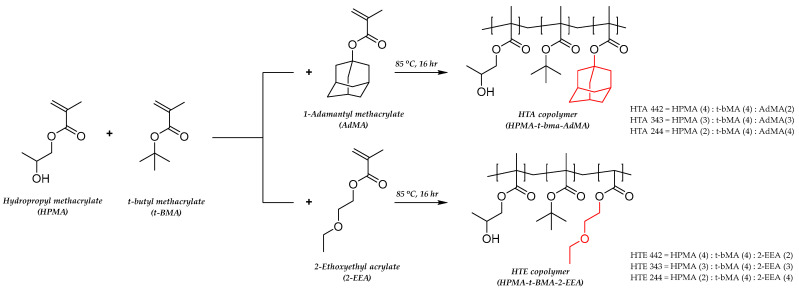
Designs of (meth)acrylate-based polymers using free radical polymerization; HTA (HPMA-t-BMA-AdMA), HTE (HPMA-t-BMA-2-EEA). Black: controlled groups; red: investigated groups.

To investigate the effect of the developer on (meth)acrylate-based polymers for negative photoresists, we synthesized polymers at different ratios. This approach allowed us to assess both the impact of functional groups with varying properties and the influence of the ratio of each group. The polymers were synthesized following the structure outlined in previous studies [22,25]. In the two types of (meth)acrylate polymers, HPMA was used as a polar group, t-BMA as an acid-labile group, 2-EEA as a hydrophilic group, and AdMA as a hydrophobic group. In this study, we designated the polymer containing the hydrophobic group AdMA as HTA (HPMA, t-BMA, AdMA) based on its composition, while the polymer containing the hydrophilic group 2-EEA was named HTE (HPMA, t-BMA, 2-EEA) following the same naming convention (Scheme 1).

**Figure 1:**
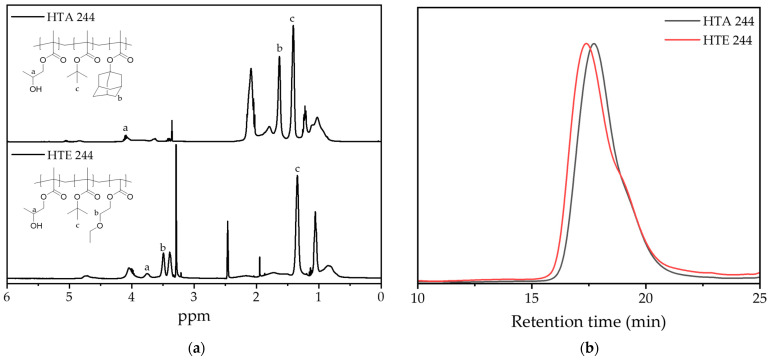
(**a**) ^1^H NMR spectra of the HTA 244 and HTE 244 polymers, (**b**) GPC data of the HTA 244 and HTE 244 polymers.

To confirm the composition of the polymers synthesized at various ratios, the characteristic proton peaks of each group were analyzed using ^1^H NMR spectra (Figure 1 and Figure S1). In the ^1^H NMR spectra of the HTA and HTE polymers, a peak at δ = 4.8 ppm, corresponding to the hydroxyl group of HPMA, and a peak at δ = 1.43 ppm, attributed to t-BMA, were observed [29,30,31]. Additionally, in the ^1^H NMR spectrum of the HTA polymer, the peak at δ = 1.6 ppm confirms the presence of AdMA (Figure 1 and Figure S1a) [32], while the peak at δ = 3.5 ppm in the HTE polymer confirms the presence of 2-EEA (Figure 1 and Figure S1b) [33,34,35]. The composition ratio of the polymer was determined through the integral values of the characteristic proton peaks (Table 1) [36,37]. In free radical polymerization, the feed ratios and the final composition ratios of the polymer can differ owing to the varying reactivity of each monomer [36,37]. However, a consistent trend in the ratio of hydrophobic to hydrophilic groups was observed in each polymer [38,39]. This trend confirms that the solubility of the polymers can be compared based on the ratio of hydrophobic to hydrophilic groups in the synthesized polymers.

The molecular weight of a polymer is an important factor that influences solubility [38]. Thus, the molecular weight is related to the solubility of TMAH in polymers. To investigate the solubility of TMAH in relation to hydrophobic or hydrophilic groups, it is essential for the molecular weights of the polymers to be similar. Sohn et al. noted that molecular weights of 8500 and 6400 are comparable [38]. Based on this, we analyzed the molecular weight ratio (6400/8500 = 0.75) to assess the similarity. The molecular weights of the polymers were determined using GPC. The GPC data indicated similar molecular weights (ratio = 0.80) for the polymers investigated (Figure 1b and Figure S2). This confirms that the difference in molecular weight does not considerably impact the solubility of the polymers [38].

### 3.2. Characterization of Polymer Matrix 

**Figure 2:**
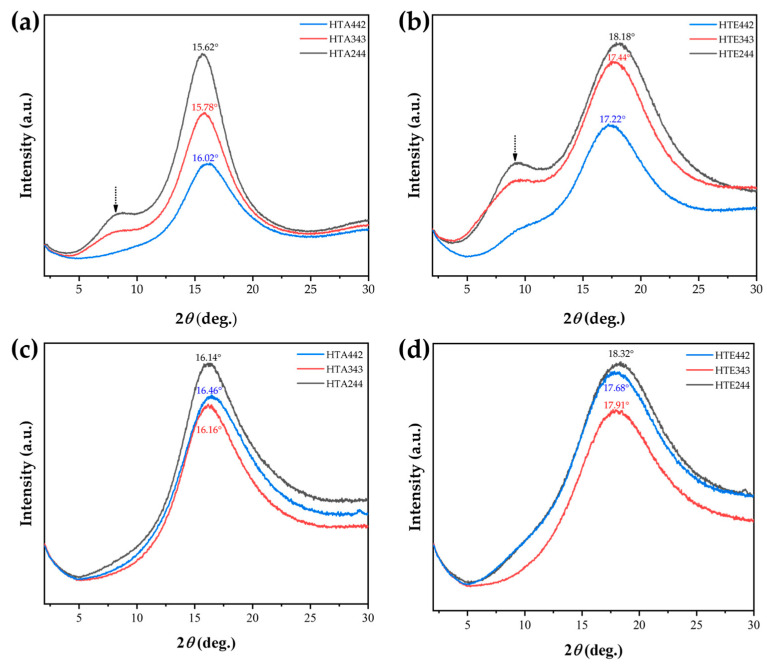
XRD spectra of the various states of ArF polymers: Unexposed (**a**) HTA; (**b**) HTE; exposed (**c**) HTA; and (**d**) HTE polymer-based photoresist film. The arrows indicate the peak of the side groups in the HTA polymer.

The distance between adjacent polymer backbones (*d*-spacing) is a key parameter related to solubility. As the d-spacing increases, solvents can more easily penetrate the polymer matrix, leading to increased solubility [40]. The d-spacing of each polymer was determined from the XRD data (Figure 2). It was calculated using Bragg’s equation (Equation (1)) from the *θ* values in the XRD spectra (Table 2). The HTA polymer displayed a characteristic diffraction peak at 2*θ* = 15.62°–16.46° (Figure 2a), while the HTE polymer exhibited a peak at 2*θ* = 17.22°–18.32° (Figure 2b). Additionally, both curves showed a secondary peak below the main diffraction peak, which corresponds to the distance between the side chains, affecting polymer interactions such as hydrophilicity. As the polymers incorporated functional groups such as AdMA and 2-EEA, the intensities of these side peaks decreased [32]. As previously mentioned, the d-spacing is influenced by the volume of the pendant groups and intermolecular forces [32,41]. The hydrophobic ring structure occupies a larger volume than the hydrophilic chain structure, resulting in a relatively wide d-spacing. These trends were observed even when the d-spacing was somewhat narrower, owing to the deprotection of *t*-BMA in the polymer after exposure (Figure 2c,d and Figure S3) [42]. In contrast, TMAH penetrates the HTE polymer with its narrower d-spacing. In HTA, the hydrophobic ring structure increases the d-spacing but reduces solubility by increasing the density between the main chains [32,43]. However, the flexible hydrophilic chain structure of HTE leads to narrower d-spacing yet contributes to relatively higher solubility [44]. These results confirm that the d-spacing of the polymer does not considerably impact its solubility in the developer.

(1) d=λ/2sinθ

**Figure 3:**
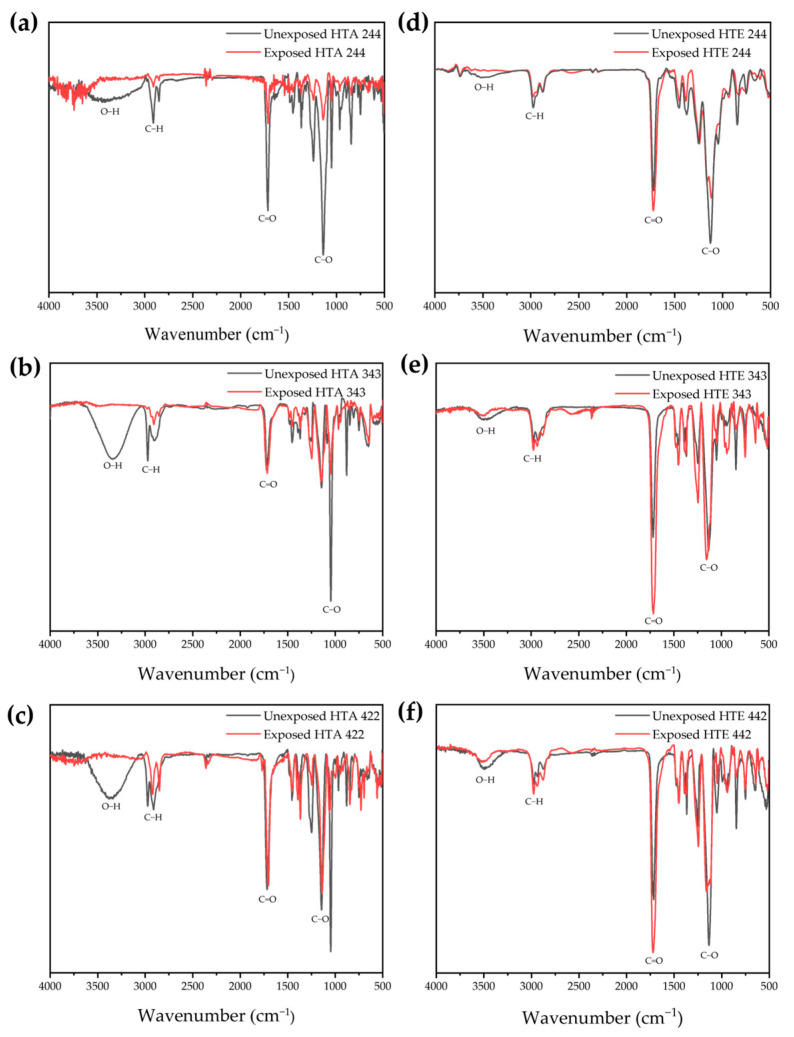
FT–IR spectra of unexposed and exposed films of (**a**–**c**) HTA 244, 343, 442 and (**d**–**f**) HTE 244, 343, 442 polymer-based photoresists.

Transesterification in the structures of HTA and HTE polymers was examined using FT–IR analysis. The broad peak between 3000 and 3700 cm^−1^, which corresponds to the hydroxyl group of HPMA, was absent after exposure (Figure 3) [45]. As confirmed in a previous study, this absence is attributed to transesterification with the hydroxy group containing the carbon chain [46]. Additionally, when the polymer is cross-linked, its solubility in the solvent decreases. To investigate this, PGMEA, which was used in the polymer synthesis, was also used. This allowed us to examine the change in solubility after the polymer was exposed and cross-linked. The solubility test performed with the exposed polymers in PGMEA showed that the polymers did not dissolve owing to the cross-linking (Figure S4). This confirms that HPMA is cross-linked by the generated acid, regardless of the presence of hydrophobic or hydrophilic groups (Figure S5).

### 3.3. Solubility of the Developer TMAH in the Copolymers 

To assess the solubility of TMAH in HTA and HTE polymer-based photoresist films, the contact angle (CA) of each film was measured. In the HTA polymer-based films, the CA increased as the proportion of hydrophobic groups increased, regardless of exposure (Figure S6). This behavior is attributed to the low affinity of the hydrophobic groups for TMAH, an aqueous alkaline developer [25]. Previous studies have indicated that films with a CA between 0 and 70 degrees are fully wetted by the solvent, while those with a CA above 70 degrees are only partially wetted [47]. In the case of HTA 442 and 343 (Figure S6d,e), t-BMA underwent deprotection after exposure to form hydroxyl groups, resulting in a CA of less than 70 degrees, which allowed TMAH penetration [48]. However, HTA 244 exhibited a CA greater than 70 degrees even after exposure (Figure S6f), suggesting that polymers with a high proportion of hydrophobic groups limit the solubility of TMAH [47].

**Figure 4:**
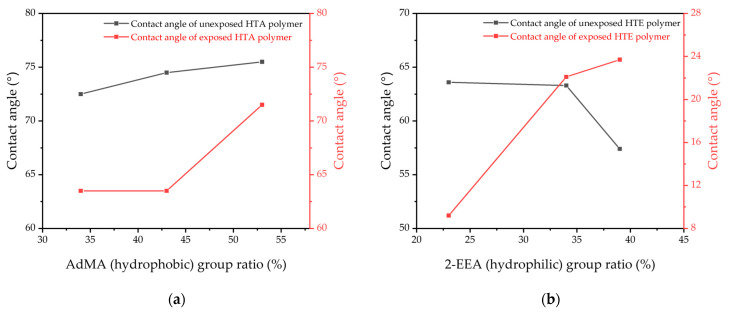
Contact angle trend of photoresist films to TMAH as a function of the ratio of (**a**) hydrophobic and (**b**) hydrophilic groups.

For unexposed HTE polymer-based photoresist films, the CA decreased as the proportion of hydrophilic groups increased (Figure S7a–c). However, for exposed films, CA increased as the proportion of hydrophilic groups increased (Figure S7d–f) [48]. This behavior is attributed to 2-EEA being more hydrophobic than the acrylic acid group formed upon exposure to the polymer [49]. Consequently, 2-EEA dominated the hydrophilic properties in the unexposed films, while the acrylic acid group dominated the hydrophilic properties in the exposed films. The change in CA in HTA and HTE polymer-based photoresist films, based on the ratio of irradiated groups, is shown in Figure 4. Our investigation of CA revealed that as the proportion of hydrophobic groups decreases and the proportion of hydrophilic groups increases, the affinity for TMAH increases, resulting in a decrease in CA. The variation ratio of the film weight upon the solvent (W_s_/W_d_) supports the results of the CA investigation (Figure S8) [50]. W_s_ represents the weight of the film after immersion in a solvent, while W_d_ denotes the weight of the dried film. To measure the variation ratio of the photoresist film, HTA and HTE were spin-coated onto the wafer at 1000 rpm for 4 min. After annealing the film at 90 °C, the weight of the coated film was recorded (W_d_). After 3 min of UV light exposure, the films were annealed for 1 min. The film was then developed in TMAH for 1 min. After blow-drying the developer remaining on the film’s surface, its weight was measured (W_s_). The results showed that the weight of the HTE polymer-based film increased after development in TMAH owing to the absorption of TMAH, driven by its hydrophilic nature. In contrast, the HTA polymer-based film retained its weight because its hydrophobicity prevented substantial absorption of TMAH.

### 3.4. Evaluation of Patterns 

**Figure 5:**
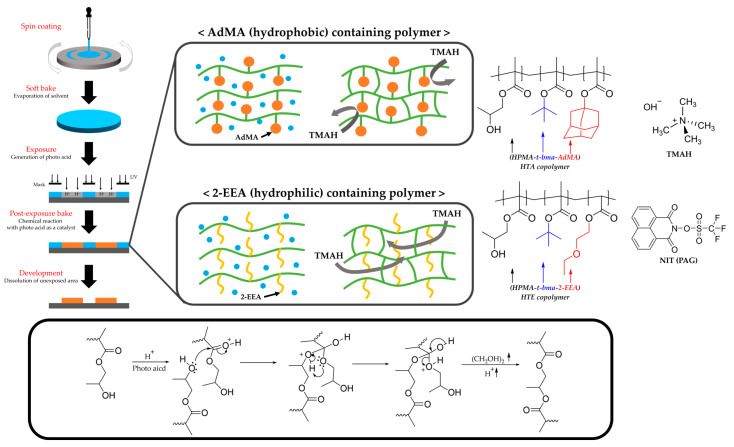
Schematic representation of the patterning process with photoresists based on HTA and HTE polymers and the diffusion process of TMAH in HTA and HTE polymers (**upper**) in relation to the cross-linking reaction outcomes (**lower**). Black: HPMA; blue: t-BMA; red: AdMA and 2-EEA.

**Figure 6:**
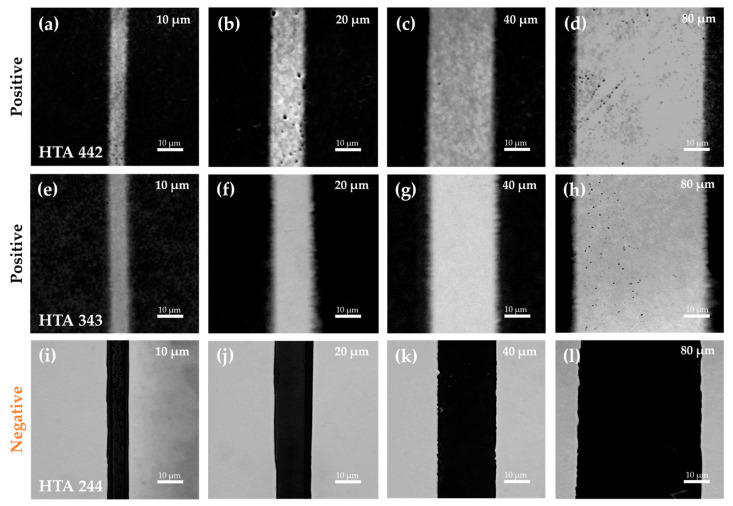
Optical microscope images of patterned photoresist films based on HTA polymer, categorized by line width: HTA 442 (10, 20, 40, and 80 μm: (**a**–**d**)); HTA 343 (10, 20, 40, and 80 μm: (**e**–**h**)); HTA 244 (10, 20, 40, and 80 μm: (**i**–**l**)).

**Figure 7:**
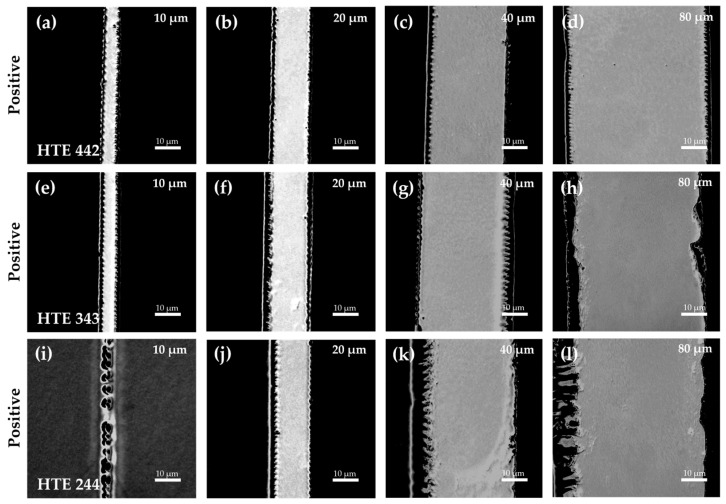
Optical microscope images of patterned photoresist films based on HTE polymer, categorized by line width: HTE 442 (10, 20, 40, and 80 μm: (**a**–**d**)); HTE 343 (10, 20, 40, and 80 μm: (**e**–**h**)); HTE 244 (10, 20, 40, and 80 μm: (**i**–**l**)).

**Figure 8:**
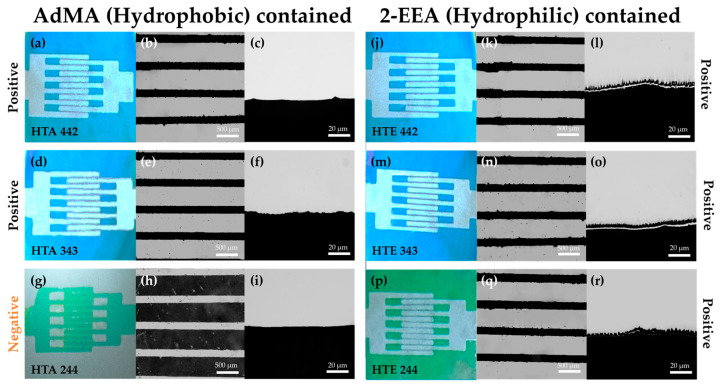
Optical microscope images of transistor patterns with photoresist based on HTE polymers at different magnifications: HTA 442 (×1, ×5, and ×100: (**a**–**c**)); HTA 343 (×1, ×5, and ×100: (**d**–**f**)); HTA 244 (×1, ×5, and ×100: (**g**–**i**)); HTE 442 (×1, ×5, and ×100: (**j**–**l**)); HTE 343 (×1, ×5, and ×100: (**m**–**o**)); HTE 244 (×1, ×5, and ×100: (**p**–**r**)).

Pattern evaluation was performed to assess the characteristics of patterns in relation to TMAH solubility in HTA and HTE polymer-based photoresists. The patterning process with the investigated photoresist polymers (HTA and HTE) and the diffusion of TMAH in the hydrophobic (HTA) and hydrophilic (HTE) polymers are depicted in Figure 5. The solubility of TMAH in the polymer affects both the development process and the swelling of the photoresist films. The variation in TMAH solubility can be observed in cross-linked polymer photoresist films after exposure. When the exposed photoresist exhibits low TMAH solubility, the cross-linked regions remain intact following development. In this film, the patterns consist of the cross-linked portions exposed by the photomask, and these patterns are referred to as negative tones. In contrast, when the exposed photoresist has solubility in TMAH, the cross-linked portions are removed during the development process, leaving behind the remaining photoresist, which forms positive-tone patterns. Various line widths (10, 20, 40, and 80 μm) were investigated to examine the surface and structure of the line patterns (Figure 6 and Figure 7). When the HTA polymer, primarily containing AdMA, was used, its hydrophobicity reduced the solubility in TMA. As a result, a negative-tone pattern was observed for the HTA 244 polymer (Figure 6a–d), which has a low solubility in TMAH. In contrast, when the AdMA ratio was lower than in HTA 244, a positive-tone pattern was observed owing to the relatively higher solubility in TMAH (Figure 6e–l). This confirms that for polymers with hydrophobic groups above a certain ratio, the solubility in TMAH does not increase after exposure, resulting in a negative-tone pattern where the exposed portions remain. However, when the HTE polymer, primarily containing 2-EEA, was used (Figure 7), the solubility in TMAH increased owing to its hydrophilic nature. As a result, a positive-tone pattern was observed under all conditions. Additionally, it was confirmed that the edge of the pattern was rougher compared to when the HTA polymer was used because the unexposed area also had a relatively high affinity for TMAH. Based on this, when HTA and HTE polymers were applied to the transistor pattern, which plays an important role in the electrical industry (Figure 8) [51], HTA 244 exhibited a negative-tone pattern (Figure 8g–i), while in other cases, a positive-tone pattern was formed (Figure 8a–f). When the HTE polymer was used (Figure 8j–r), the surface of the pattern showed signs of collapse.

## 4. Conclusions

We synthesized two types of polymers to compare their effect on TMAH in (meth)acrylate-based negative photoresists. To investigate the role of the adamantyl monomer in controlling solubility in the developer, 2-EEA and AdMA were used to synthesize hydrophilic and hydrophobic polymers, respectively. In both polymers, HPMA served as the polar group of the photoresist, and t-BMA was included as the acid-labile group. Composition trends were confirmed through ^1^H NMR analysis of the polymers synthesized at various ratios, and the relationship between feed ratio and composition ratio was also examined. To control potential variables affecting developer solubility, the molecular weight related to solubility was assessed using GPC data, and the d-spacing of the polymer, which influences solubility, was determined through XRD spectra. This analysis confirmed that the hydrophilic and hydrophobic properties of the polymer were primarily governed by the functional groups rather than other variables. Additionally, FT–IR analysis revealed a broad peak for the hydroxyl group, indicating the negative tone of the photoresist owing to the transesterification reaction of HPMA during exposure. When hydrophobic AdMA was the main component of the polymer, the CA remained consistent even with an increase in the hydroxyl group, resulting from deprotection after exposure. In contrast, when hydrophilic 2-EEA was incorporated into the polymer, the CA increased in proportion to the amount of 2-EEA owing to its lower hydrophilicity compared to the hydroxyl group. Polymers containing both hydrophilic and hydrophobic groups are typically used in positive photoresists. However, a polymer is used as a negative photoresist when hydrophobic groups make up more than 50% of the polymer composition. This result confirms that the solubility of TMAH in negative photoresist polymers can be controlled by a higher proportion of hydrophobic groups. Based on these results, this study demonstrates that adamantyl monomers effectively function as aliphatic groups to regulate the solubility of (meth)acrylate polymers in developing solutions.

## Figures and Tables

**Table 1:** Molar feed ratio and copolymer composition of HTA and HTE polymers at various ratios determined by ^1^H NMR, and weight average molecular weight (M_n_) and polydispersity (PDI) determined by GPC.

Polymer	Molar Feed Ratios (Copolymer Composition)				M_n_ (g/mol)	PDI (*Đ*)
	HPMA	t-BMA	AdMA	2-EEA		
HTA442	4 (4.71)	4 (4)	2 (2.43)	-	10,090	1.82
HTA343	3 (3.62)	4 (4)	3 (3.36)	-	11,413	1.67
HTA244	2 (2.22)	4 (4)	4 (4.91)	-	11,925	1.59
HTE442	4 (2.87)	4 (4)	-	2 (2.43)	11,028	1.74
HTE343	3 (1.94)	4 (4)	-	3 (3.06)	11,413	1.71
HTE244	2 (1.41)	4 (4)	-	4 (3.91)	12,605	1.67

**Table 2:** XRD peak positions (2*θ*) and d-spacing values of photoresist films with different polymers.

Polymer	2*θ* ^1^ (˚)	2*θ* ^2^ (˚)	d-Spacing ^1^ (Å)	d-Spacing ^2^ (Å)
HTA442	16.02	16.46	5.47	5.38
HTA343	15.78	16.16	5.61	5.48
HTA244	15.62	16.14	5.67	5.49
HTE442	17.22	17.68	5.15	5.01
HTE343	17.44	17.91	5.08	4.95
HTE244	18.18	18.32	4.87	4.84

^1^ Unexposed under the light source, ^2^ Exposed under the light source.

## Data Availability

The original contributions presented in the study are included in the article and Supplementary Material, further inquiries can be directed to the corresponding author.

## Supplementary Materials

The following supporting information can be downloaded at: www.mdpi.com/article/10.3390/ma18020381/s1, Figure S1: ^1^H NMR spectra of (a) HTA and (b) HTE polymers; Figure S2: GPC data for the (a) HTA and (b) HTE polymers at various ratios; Figure S3: Scheme illustrating the photoacid generation reaction via photolysis of N-((trifluoromethyl sulfonyl)oxy)-5-norbornene-2,3-dicarboximide (NDI) and the deprotection reaction of t-BMA; Figure S4: Image of a polymer in PGEMEA solution cross-linked by photoacids upon exposure to light; Figure S5: Scheme depicting the cross-linking reaction via transesterification of HPMA by photoacid; Figure S6: Images of TMAH contact angles on HTA polymer-based photoresist films synthesized at various ratios: (a–c) unexposed HTA 442, 343, and 244 films; (d–f) exposed HTA 442, 343, and 244 films; Figure S7: Images of TMAH contact angles on HTE polymer-based photoresist films synthesized at various ratios: (a–c) unexposed HTE 442, 343, and 244 films; (d–f) exposed HTE 442, 343, and 244 films; Figure S8: Graph of weight ratio (W_s_/W_d_) of HTA and HTE polymer-based films obtained through TMAH immersion.
