# Supplementary material for: Controlling the Dissolution Behavior of (Meth)acrylate-Based Photoresist Polymers in Tetramethylammonium Hydroxide by Introducing Adamantyl Groups
Source: Materials (Basel). 2025 Jan 15;18(2):381. doi: 10.3390/ma18020381 (PMC11766972; doi:10.3390/ma18020381)
Supplement: Supplementary file 1 [file materials-18-00381-s001.zip › materials-3108148-supplementary.pdf]

materials

MDPI

Supplementary Materials

Controlling the Dissolution Behavior of (Meth)acrylate-Based Photoresist Polymers in Tetramethylammonium Hydroxide by Introducing Adamantyl Groups

Jinyoung Kim, Choong-Jae Lee, Dong-Gun Lee, Geon-Ho Lee, Jayoung Hyeon, Yura Choi and Namchul Cho *

Department of Energy Engineering, Soonchunhyang University, 22 Soonchunhyang-ro, Asan 31538, Republic of Korea; kjy1624@sch.ac.kr (J.K.); cndwolee1397@sch.ac.kr (C.-J.L.); alclsro6202@naver.com (D.-G.L.); sinb0603@sch.ac.kr (G.-H.L.); jayoung@sch.ac.kr (J.H.); bnbn3238@sch.ac.kr (Y.C.)

* Correspondence: chon7@sch.ac.kr

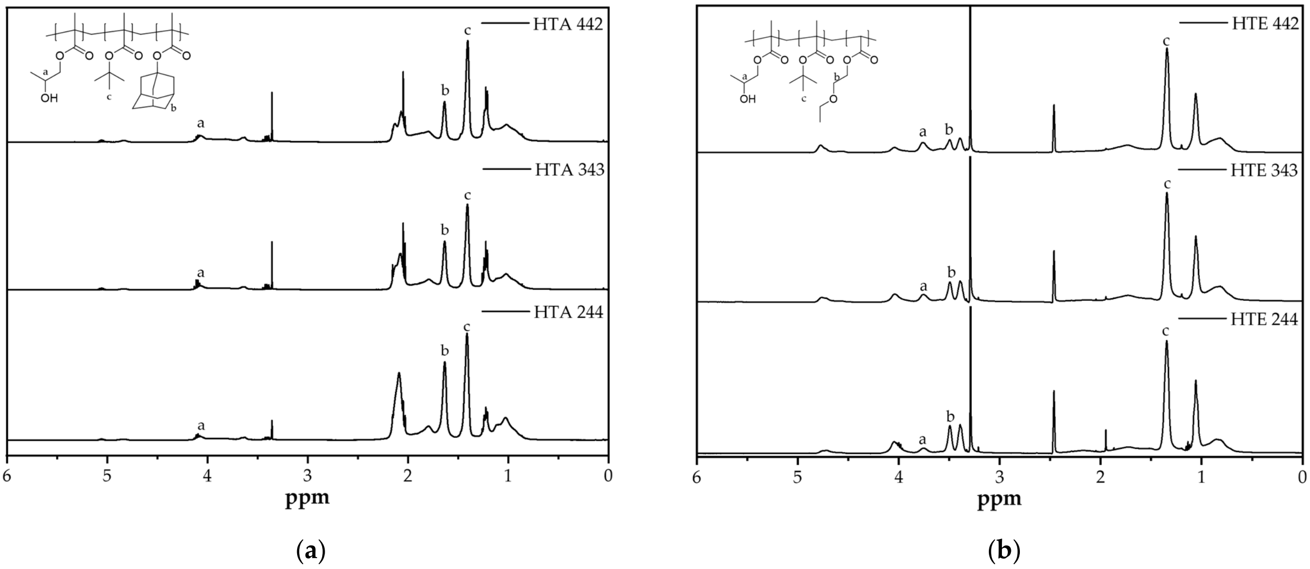

Figure S1. ^{1}H NMR spectra of (a) HTA and (b) HTE polymers.

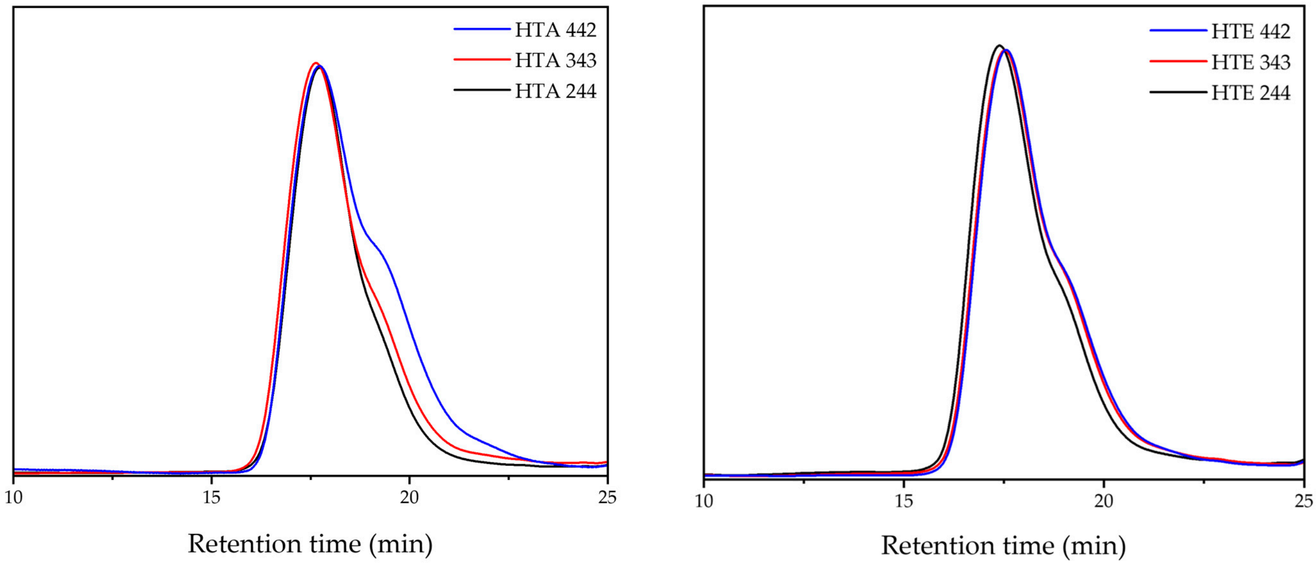

(**a**) (**b**)

Figure S2. GPC data for the (a) HTA and (b) HTE polymers at various ratios.

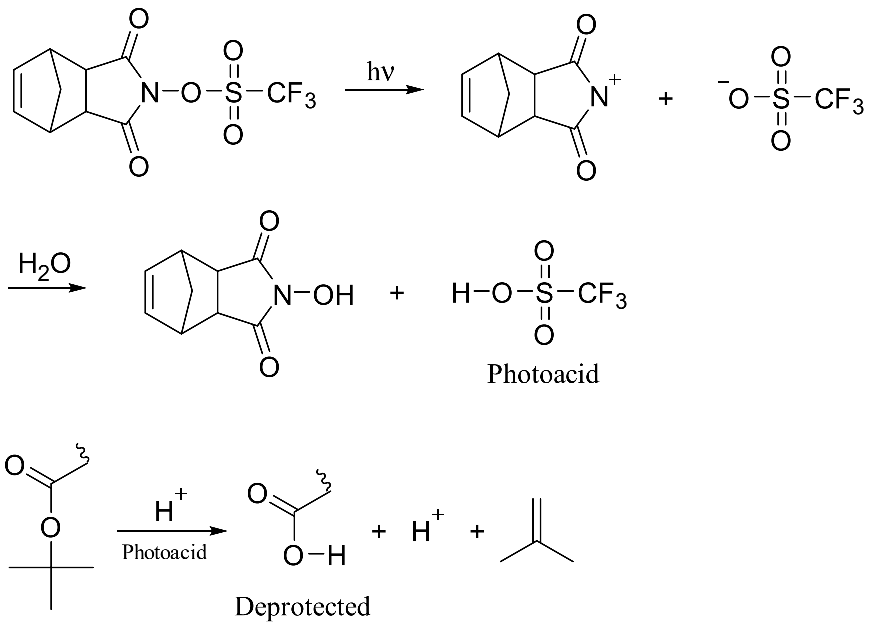

Figure S3. Scheme illustrating the photoacid generation reaction via photolysis of N-((trifluoromethyl sulfonyl)oxy)-5-norbornene-2,3-dicarboximide (NDI) and the deprotection reaction of t-BMA.

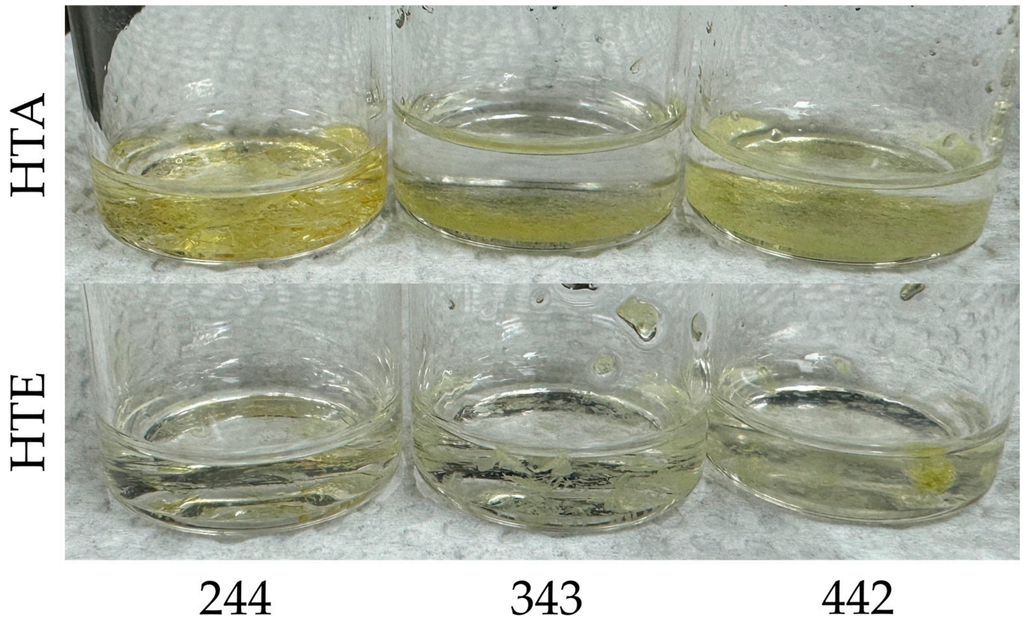

Figure S4. Image of a polymer in PGEMEA solution cross-linked by photoacids upon exposure to light.

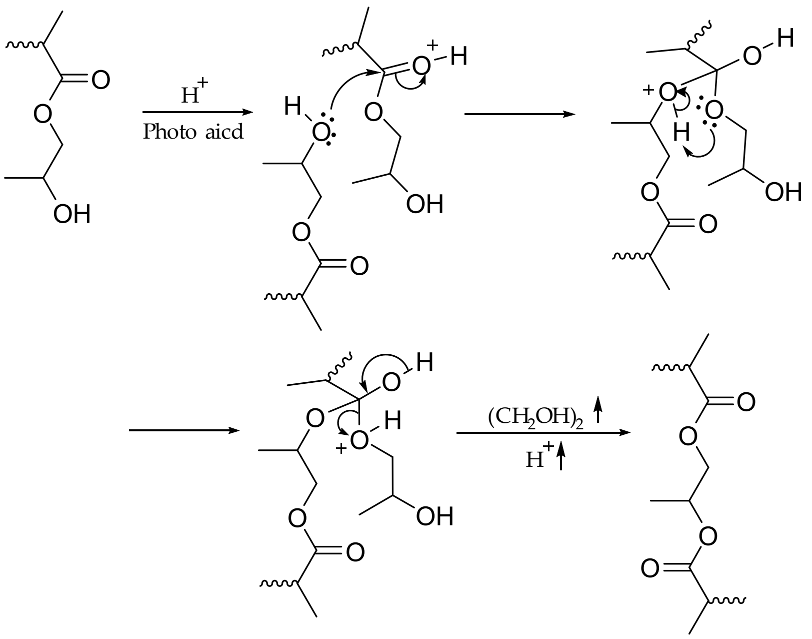

Figure S5. Scheme depicting the cross-linking reaction via transesterification of HPMA by photoacid.

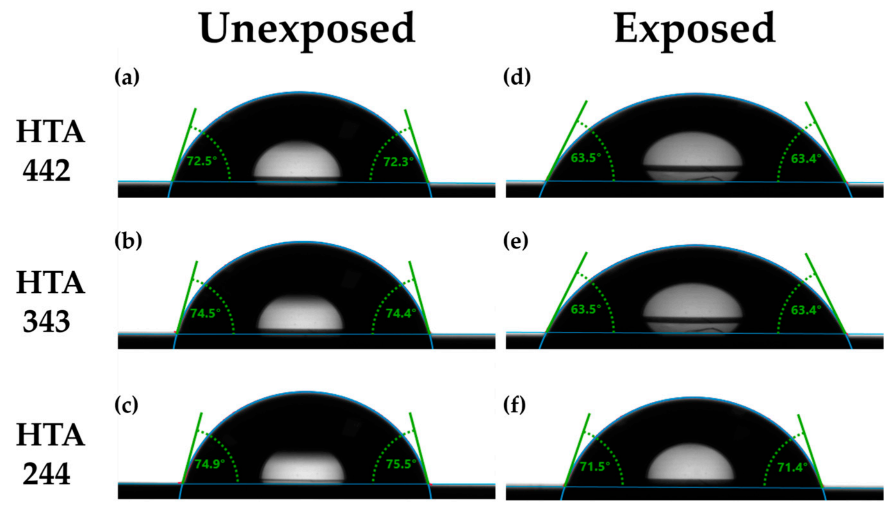

Figure S6. Images of TMAH contact angles on HTA polymer-based photoresist films synthesized at various ratios: (a–c) unexposed HTA 442, 343, and 244 films; (d–f) exposed HTA 442, 343, and 244 films.

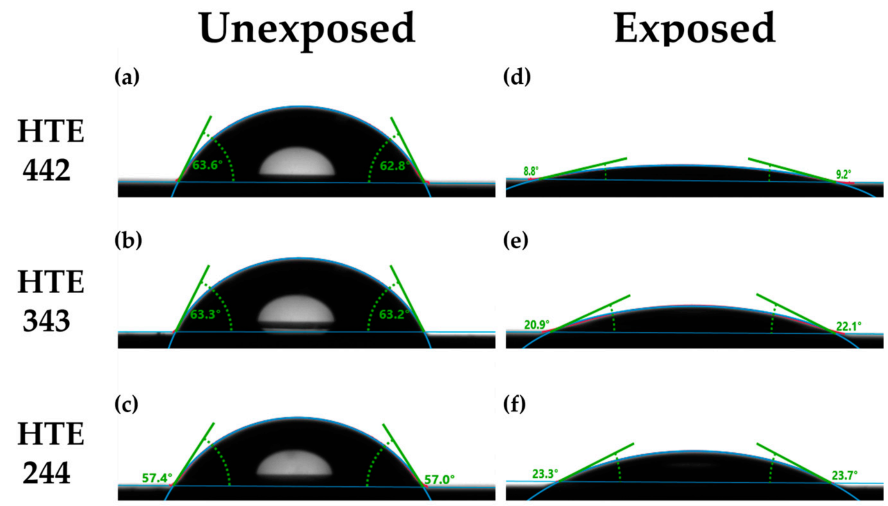

Figure S7. Images of TMAH contact angles on HTE polymer-based photoresist films synthesized at various ratios: (a–c) unexposed HTE 442, 343, and 244 films; (d–f) exposed HTE 442, 343, and 244 films.

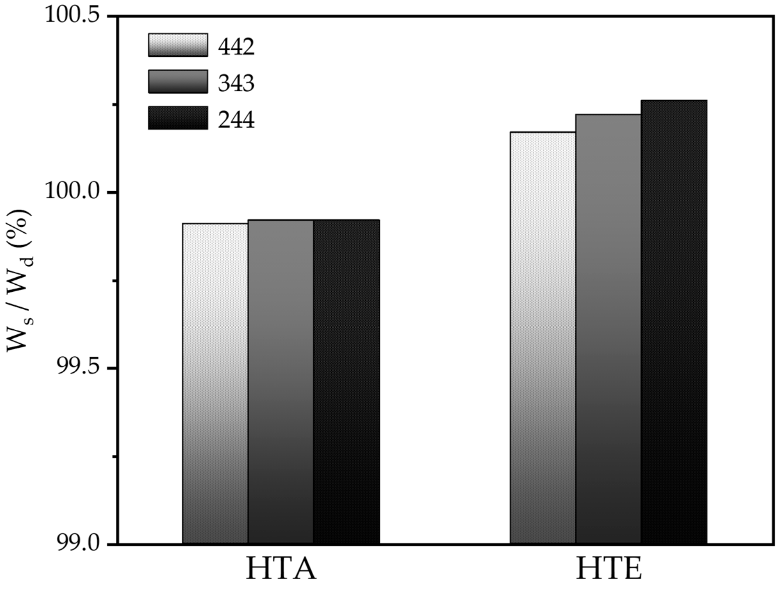

Figure S8. Graph of weight ratio (W_s / W_d) of HTA and HTE polymer-based films obtained through TMAH immersion.
